# Differential Mitochondrial Bioenergetics in Neurons and Astrocytes Following Ischemia-Reperfusion Injury and Hypothermia

**DOI:** 10.3390/biomedicines12081705

**Published:** 2024-08-01

**Authors:** Santiago J. Miyara, Koichiro Shinozaki, Kei Hayashida, Muhammad Shoaib, Rishabh C. Choudhary, Stefanos Zafeiropoulos, Sara Guevara, Junhwan Kim, Ernesto P. Molmenti, Bruce T. Volpe, Lance B. Becker

**Affiliations:** 1 Elmezzi Graduate School of Molecular Medicine, Manhasset, NY 11030, USA; 2 Feinstein Institutes for Medical Research, Manhasset, NY 11030, USA; 3 Department of Emergency Medicine, Northwell Health, Manhasset, NY 11030, USA; 4 Department of Surgery, Renown Health, Reno, NV 89502, USA; 5 Department of Emergency Medicine, Kindai University Faculty of Medicine, Osaka 589-8511, Japan

**Keywords:** hypothermia, ischemia-reperfusion injury, neurons, astrocytes, neuroprotection, cardiac arrest

## Abstract

The close interaction between neurons and astrocytes has been extensively studied. However, the specific behavior of these cells after ischemia-reperfusion injury and hypothermia remains poorly characterized. A growing body of evidence suggests that mitochondria function and putative transference between neurons and astrocytes may play a fundamental role in adaptive and homeostatic responses after systemic insults such as cardiac arrest, which highlights the importance of a better understanding of how neurons and astrocytes behave individually in these settings. Brain injury is one of the most important challenges in post-cardiac arrest syndrome, and therapeutic hypothermia remains the single, gold standard treatment for neuroprotection after cardiac arrest. In our study, we modeled ischemia-reperfusion injury by using in vitro enhanced oxygen-glucose deprivation and reperfusion (eOGD-R) and subsequent hypothermia (HPT) (31.5 °C) to cell lines of neurons (HT-22) and astrocytes (C8-D1A) with/without hypothermia. Using cell lysis (LDH; lactate dehydrogenase) as a measure of membrane integrity and cell viability, we found that neurons were more susceptible to eOGD-R when compared with astrocytes. However, they benefited significantly from HPT, while the HPT effect after eOGD-R on astrocytes was negligible. Similarly, eOGD-R caused a more significant reduction in adenosine triphosphate (ATP) in neurons than astrocytes, and the ATP-enhancing effects from HPT were more prominent in neurons than astrocytes. In both neurons and astrocytes, measurement of reactive oxygen species (ROS) revealed higher ROS output following eOGD-R, with a non-significant trend of differential reduction observed in neurons. HPT after eOGD-R effectively downregulated ROS in both cells; however, the effect was significantly more effective in neurons. Lipid peroxidation was higher after eOGD-R in neurons, while in astrocytes, the increase was not statistically significant. Interestingly, HPT had similar effects on the reduction in lipoperoxidation after eOGD-R with both types of cells. While glutathione (GSH) levels were downregulated after eOGD-R in both cells, HPT enhanced GSH in astrocytes, but worsened GSH in neurons. In conclusion, neuron and astrocyte cultures respond differently to eOGD-R and eOGD-R + HTP treatments. Neurons showed higher sensitivity to ischemia-reperfusion insults than astrocytes; however, they benefited more from HPT therapy. These data suggest that given the differential effects from HPT in neurons and astrocytes, future therapeutic developments could potentially enhance HPT outcomes by means of neuronal and astrocytic targeted therapies.

## 1. Introduction

Cardiac arrest (CA) has an annual toll of 500,000 human lives every year in the United States [1]. Despite significant improvements in the clinical management of cardiopulmonary resuscitation and post-resuscitation efforts, neurological injury from global ischemia-reperfusion injury represents a major cause of mortality and morbidity among CA survivors [2]. Although the resuscitation efforts after CA are oriented to restore cerebral blood flow through the return of spontaneous circulation (ROSC), a compelling body of evidence has shown that reperfusion damage can indeed surpass the initial injury caused by the ischemic insult [3]. In the last 20 years, numerous studies have shown that mitochondria function and dysfunction play a fundamental role following ischemia-reperfusion injury (IRI) [4,5,6]. Despite recent concerns raised by the international, multicentric TTM2 trial, therapeutic hypothermia still represents the only widely accepted treatment for neuroprotection after ROSC [7,8]. In cardiac arrest, the neuroprotective mechanisms behind hypothermia are still under investigation; however, a growing body of scientific research has revealed that hypothermia can modulate mitochondrial bioenergetics, dynamics, redox status, and mitochondrial-dependent cell death [9,10,11,12,13]. In 2016, Hayakawa and collaborators discovered a novel mechanism in astrocytic–neuronal symbiosis, based on mitochondrial transfer from astrocytes to neurons during experimental stroke in mice. More specifically, they demonstrated that in astrocytes, a calcium-dependent mechanism involving CD38 and cyclic ADP signaling regulates mitochondrial particles releasing to neurons, in which mitochondrial uptake was associated with enhanced cell survival [14]. These novel findings fostered an interest in mitochondrial transference and transplantation as therapeutic targets in conditions associated with IRI; however, the specific biology and differential effect of HPT after IRI in neurons and astrocytes have not been properly characterized [6]. To gain insights into these differential cellular responses, in this study, we modeled in vitro IRI in single cultures of neurons (HT-22) and astrocytes (C8-D1A) by using an enhanced protocol of 6 h oxygen-glucose deprivation and 20 h reperfusion (eOGD-R) and hypothermia [15]. The eOGD-R + hypothermia (31.5 °C) group was compared with eOGD-R + normothermia (37 °C) and the controls. Cell lysis (LDH; lactate dehydrogenase) was used as a marker of cell viability and membrane disruption. Total ATP output was measured to estimate bioenergetics and oxidative phosphorylation. Global reactive oxygen species (ROS) were quantified to understand the overall redox status changes while lipid peroxidation was determined as a surrogate marker of oxidative stress. Glutathione (GSH), which is a key physiological free radical scavenger, was determined to enhance our insights into the redox status after eOGD-R and HPT.

## 2. Materials and Methods

### 2.1. Cell Culture

Neurons were cultured from the immortalized cell line HT-22 (Sigma-Aldrich, St. Louis, MO, USA, Catalog #SCC129), originated in the hippocampus of mice. Astrocytes were cultured from the immortalized cell line C8-D1A (ATCC, Manassas, VA, USA, Catalog #CRL-2541), originated from mice. Both cell lines were grown as single cultures in 6 cm Petri dishes at standard conditions (humidified incubator, 37 °C and 5% CO_2_) and subcultured not more than five times using trypsin-EDTA (0.05%) (Gibco, Grand Island, NY, USA, Catalog #25-300-054). Cell medium (Dulbecco’s modified Eagle’s medium, Thermo Fisher, Waltham, MA, USA, Catalog #11995073) was replaced every 48 h, and subcultures were made after reaching 80% of confluence. Cell medium was enriched with 10% fetal bovine serum (FBS) (Gibco, USA, Catalog #16-140-071) and 1% penicillin/streptomycin (P/S) (Gibco, USA, Catalog #15140122).

### 2.2. In Vitro Ischemia-Reperfusion Injury

In vitro ischemia-reperfusion injury was modeled by combining severe hypoxia and chemical ischemia (eOGD-R, i.e., enhanced OGD-R protocol); an oxygen concentrator (Bulldog Bio, Portsmouth, NH, USA, nBIONIX Hypoxic Cell Culture Kit) was used to reduce oxygen concentrations from 21% to 1.5% while chemical ischemia was accomplished by replacing the regular medium with nutritionally depleted medium (no glucose, no pyruvate, no glutamine, no FBS, no phenol red, and no P/S) (Thermo Fisher, Catalog #A1443001). The ischemia protocol lasted 6 h, while reperfusion 20 h. Reperfusion was carried out by returning cells to standard conditions (regular DMEM medium, humidified incubation at 37 °C, CO_2_ 5%, and O_2_ 21%). 

### 2.3. Hypothermia Protocol

Hypothermia treatment was applied throughout the length of reperfusion (20 h) by using a humidified cell incubator (Thermo Scientific™, Heracell™ 150i CO_2_ Incubator) thermoregulated at 31.5 °C. The temperature was corroborated by a double-probe system to ensure consistent values of moderate hypothermia [16].

### 2.4. Cell Lysis and Viability

Lactate dehydrogenase (LDH) measurements were determined by the CytoTox 96^®^ Non-Radioactive Cytotoxicity Assay (Promega, Madison, WI, USA, Catalog #G1780), following the standard manufacturer’s protocol described below. Cells were seeded on 96-well, white microplates (Corning, Somerville, MA, USA, Catalog #353296) for 48 h before conducting eOGD-R experimentation. Each well was seeded with approximately 9000 cells, and background wells were used for subtraction calculation in the experimental wells. After the completion of insult (eOGD-R) and treatment (hypothermia), 50 uL of media from each well was transferred to a fresh 96-well plate, 50 uL of restituted CytoTox 96^®^ reagent was added concomitantly to each well, and posteriorly incubated for 30 min protected from light in an opaque environment at room temperature. When incubation was completed, 50 uL of Stop Solution was concomitantly added to each well. Calibrated multichannel pipettes were used to ensure equal and timely exposure to reagents in all wells and groups (eOGD-R, hypothermia, and controls). The colorimetric absorbance readings from the microplates were performed at a wavelength of 490 nm using a calibrated spectrophotometer (Infinite^®^ 200 Pro, Tecan, Männedorf, Switzerland). The experiment was repeated three times to demonstrate reproducibility. Sampling size is presented in the figure. The CytoTox 96^®^ Non-Radioactive Cytotoxicity Assay did not include the LDH standardized solution for standard curve determination; therefore, data interpretation was based on the comparison with the controls, and do not represent quantitative LDH values.

### 2.5. Bioenergetics

Adenosine triphosphate (ATP) was quantified by the CellTiter-Glo^®^ 2.0 (Promega, Catalog #G9242) using the standard manufacturer’s protocol. Luminescence signaling was determined using a luminescent Spark^®^ microplate reader (Tecan), with an integration time of 1 s per well. Cells were seeded on 96-well, white plates (Corning, Catalog #353296) for 48 h before conducting eOGD-R experimentation. Each well was seeded with approximately 9000 cells. No background wells were required as per the manufacturer’s protocol and our internal pilot studies. CellTiter-Glo^®^ 2.0 reagent was thawed in a 22 °C water bath. CellTiter-Glo^®^ 2.0 was mixed gently by inverting the content 10 times. Once the eOGD-R experiments were completed, we added 100 uL of CellTiter-Glo^®^ 2.0 reagent to each well using a calibrated multichannel pipette, ensuring equal and timely exposure to reagents in all wells and groups (eOGD-R, hypothermia, and controls). The experiment was repeated three times to demonstrate reproducibility. Sampling size is presented in the figure. The CellTiter-Glo^®^ 2.0 Kit did not include the ATP standardized solution for standard curve determination; therefore, data interpretation was based on the comparison with the controls, and do not represent quantitative ATP values. The CellTiter-Glo^®^ 2.0 Assay provides a method to determine the number of viable cells in cultures by quantitating ATP present in cells, which serves as a surrogate of metabolically active cells.

### 2.6. Reactive Oxidative Species and Lipid Peroxidation Measurement

Global measurement of reactive oxygen species (ROS) production was conducted by the fluorogenic probe CellROX™ Deep Red Reagent (Thermo Fisher Scientific, Catalog #C10422) following the standard procedures indicated by the manufacturer. Lipid peroxidation was assessed by the Image-iT^®^ Lipid Peroxidation Kit (Thermo Fisher Scientific, Catalog #C10445). A S3e Cell Sorter (Bio-Rad^®^, Hercules, CA, USA) was used for cell sorting. The experiments were repeated twice. Sampling size is presented in the figure. For the ROS and lipid peroxidation experiments, the cells were seeded in 6-well microplates at ~500,000 cells/well 48 h before conducting the eOGD-R experimentation. Experimental procedures and setup were equivalent for both the ROS and lipid peroxidation experiments.

For the ROS experiments, after the completion of the eOGD-R and hypothermia experiments, the cells were stained with 5 μM of CellROX^®^ Deep Red Reagent and incubated at 37 °C for 30 min. Subsequently, the medium was removed and the cells were washed in phosphate-buffered saline (PBS) three times, before immediate analysis using a calibrated S3e Cell Sorter (Bio-Rad^®^) set up as a flow cytometry reader. CellROX Deep Red is a cell-permeable dye that is non-fluorescent while in a reduced state, which exhibits excitation/emission at 640/665 nm following oxidation. For the lipid peroxidation experiments, after the completion of the eOGD-R and hypothermia experiments, an Image-iT^®^ Lipid Peroxidation Sensor was added to a final concentration of 10 µM to the cells. Following an incubation period of 30 min at 37 °C, the media were removed and the cells washed three times with PBS. Posteriorly, fluorescence was measured using a S3e Cell Sorter (Bio-Rad^®^). For lipid peroxidation, the Image-iT™ Lipid Peroxidation Kit facilitates the identification of lipid peroxidation in cells by utilizing the oxidation of the BODIPY™ 581/591 C11 reagent. Fluorescence was read at two separate wavelengths: one at an excitation/emission of 581/591 nm (Texas Red^®^ filter set, Thermo Fisher Scientific, Catalog #C10445) for the reduced dye, and the other at an excitation/emission of 488/510 nm (traditional FITC filter set, Thermo Fisher Scientific, Catalog #C10445) for the oxidized dye. The BODIPY™ 581/591 C11 reagent spreads across cell membranes, and upon reacting with lipid hydroperoxides, exhibits a change in fluorescence emission peak from approximately 590 nm to about 510 nm. Therefore, the ratio of the emission fluorescence intensities at 590 nm to 510 nm provides the read-out for lipid peroxidation in cells.

### 2.7. Glutathione Determination

Glutathione was quantified by the GSH-Glo™ Glutathione Assay (Promega, Catalog #V6911), as instructed by the manufacturer’s protocol. Cells were seeded on 96-well, white microplates (Corning, Catalog #353296) for 48 h before conducting the eOGD-R experimentation. After completion of the eOGD-R and hypothermia experiments, the medium was removed from the plate containing the samples, and luciferin-NT and glutathione S-transferase were added to GSH-Glo™ Reaction Buffer to create the GSH-Glo™ Reagent, which was then added (100 uL) to each well on the microplate. After a 30 min incubation at room temperature, reconstituted Luciferin Detection Reagent was added (100 uL) to each well on the microplate. Following a 15 min incubation, luminescence was measured using a luminometer Spark^®^ microplate reader (Tecan) with an integration time of 1 s per well. The experiment was repeated twice. Sampling size is presented in the figure. Background signaling was subtracted from all groups. The GSH-Glo™ Glutathione Assay is a luminescence-based approach capable of detecting glutathione; it works on the basis of converting a luciferin derivative into luciferin when glutathione is present, facilitated by the enzyme glutathione S-transferase. The resulting signal, originated by a coupled reaction with firefly luciferase, is proportional to the amount of glutathione in the cells. The GSH-Glo™ Glutathione experiments were conducted without a standard curve; therefore, data interpretation was based on the comparison with the controls, and do not represent quantitative glutathione values.

### 2.8. Statistical Analysis

The data generated were analyzed through the internal application and algorithms from GraphPad Prism 8 Edition software. Normal distribution was assessed by the Shapiro–Wilk test. Equal variances assessment was conducted by the Brown–Forsythe test and homoscedasticity plots. Parametric testing, specifically the analysis of variance (ANOVA), was performed when the data successfully passed tests for gaussian distribution and homogeneity of variance. Welch’s t-test was indicated for comparison between two groups with criteria for normal distribution. Measurements from individual cell cultures were performed in triplicate. Data are presented as the mean ± SEM. The statistical analysis of the results were performed by one-way ANOVA followed by Tukey’s test for multiple comparisons (post hoc analysis). Cross comparison of eOGD-R and hypothermia on neurons and astrocytes was accomplished by two-way ANOVA. Cell type (neurons and astrocytes) and interventions (controls, eOGD-R, and hypothermia) were declared as independent variables and read-outs (LDH, ATP, ROS, LPO, glutathione) as dependent variables. During cross-comparison analysis, controls were paired with eOGD-R, while eOGD-R was paired with hypothermia. Sidak’s test was conducted when multiple comparisons were required. In all cases, a *p*-value ≤ 0.05 was considered to be statistically significant. Notations on the level of statistical significance are presented as ns, *,**,***,**** for *p* levels of statistical significance >0.05, ≤0.05, ≤0.01, ≤0.001, ≤0.0001, respectively.

## 3. Results

**Figure 1:**
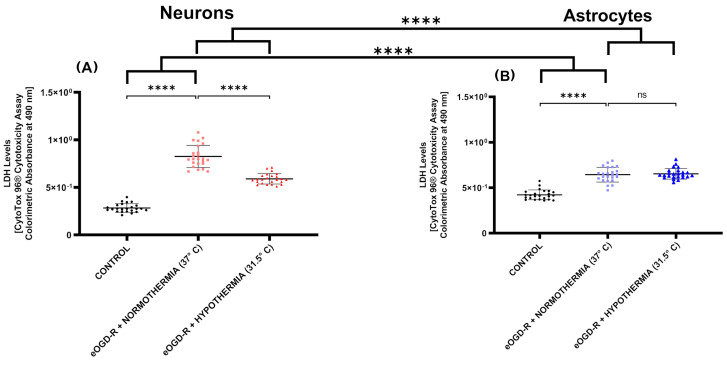
(**A**) In neurons, 6 h ischemia and 20 h reperfusion protocol in vitro (eOGD-R) caused significant cell lysis as measured by LDH (*p*-value ≤ 0.0001). Hypothermia (31.5 °C) treatment for 20 h significantly protected neurons against eOGD-R induced cell lysis, as shown by LDH downregulation (*p*-value ≤ 0.0001). (**B**) In astrocytes, 6 h + 20 h eOGD-R caused significant cell lysis as measured by LDH (*p*-value ≤ 0.0001). Hypothermia (31.5 °C) treatment for 20 h did not cause any significant effect on astrocytes against eOGD-R induced cell lysis (*p*-value = 0.86). Overall, neurons showed higher susceptibility to ischemia-reperfusion insults versus astrocytes (*p*-value ≤ 0.0001); however, only neurons showed cytoprotection from hypothermia treatment (*p*-value ≤ 0.0001).

***In neurons, eOGD-R resulted in higher LDH leakage compared with astrocytes*.** Studies have shown that not all organs are equally susceptible to IRI, nevertheless, the cell differences in neuronal and astrocytic damage elicited by an eOGD-R protocol have not been fully characterized. Thus, we investigated whether an eOGD-R protocol (6 h ischemia, 20 h reperfusion) in neurons and astrocytes could result in different degrees of membrane disruption and LDH leakage. We found that eOGD-R increased the LDH levels in both neurons and astrocytes, however, the relative increase in LDH was considerably higher in neurons compared with astrocytes (Figure 1A,B). Specifically, in neurons, LDH significantly increased 2.91 times compared with the controls, while in astrocytes, LDH increased by 1.52-fold relative to the controls (*p*-value < 0.0001).

***In neurons, hypothermia following eOGD-R decreased LDH release, while in astrocytes, no significant effect was observed.*** Compared with neurons, the current scientific literature deems astrocytes as a more resilient cell phenotype following IRI. Nevertheless, the extent of differential benefits in these two cells, as a consequence of hypothermia after IRI, remains unclear. We employed a hypothermia protocol (31.5 °C) following the eOGD (6 h) insult, lasting 20 h concomitantly with the whole reperfusion protocol. Compared with astrocytes, neurons experienced significant cytoprotection from hypothermia, as per the decreased LDH release (Figure 1A,B, *p*-value ≤ 0.0001). Hypothermia significantly buffered the LDH increase when compared with normothermia with a fold increase of 2.08 and 2.91, respectively (Figure 1A, *p*-value ≤ 0.0001). In astrocytes, the same eOGD-R and hypothermia protocol did not reveal any significant benefit compared with the normothermia group (Figure 1B, *p*-value < 0.86).

**Figure 2:**
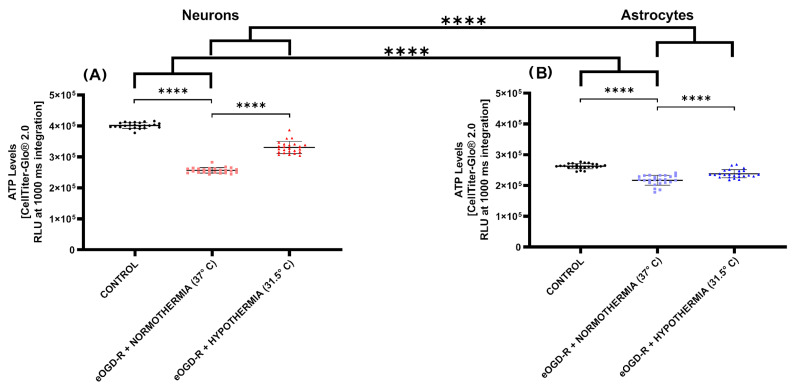
(**A**) In neurons, 6 h ischemia and 20 h reperfusion protocol in vitro (eOGD-R) caused significant ATP depletion versus the control (*p*-value ≤ 0.0001). Hypothermia (31.5 °C) treatment significantly protected neurons against eOGD-R insult, as shown by higher ATP levels compared with the normothermia group (*p*-value ≤ 0.0001). Units expressed as relative light units (RLUs). (**B**) In astrocytes, 6 h + 20 h normothermia eOGD-R caused significant ATP depletion versus the controls (*p*-value ≤ 0.0001). Hypothermia (31.5 °C) treatment for 20 h significantly protected astrocytes against eOGD-R insult, as shown by higher ATP levels compared with the normothermia group (*p*-value ≤ 0.0001). Compared with astrocytes, neurons presented the highest susceptibility for ATP depletion after eOGD-R (*p*-value ≤ 0.0001); however, hypothermia ameliorated ATP deficit most consistently in neurons (*p*-value ≤ 0.0001). Units expressed as RLU.

***Enhanced OGD-R caused higher ATP depletion in neurons than in astrocytes.*** While an exhaustive number of studies have shown that the ATP depletion crisis is a consistent hallmark of IRI pathophysiology, little has been described on how the eOGD-R protocol causes differential ATP deficit in neurons and astrocytes. Our ATP luminescent quantification revealed that in a significant fashion, eOGD-R caused downregulation in ATP production in both neurons and astrocytes (Figure 2A,B, *p*-value ≤ 0.0001). However, when compared with the controls, the observed effect was more pronounced in neurons than in astrocytes, with a fold decrease of 1.56 and 1.21, respectively (Figure 2A,B, *p*-value ≤ 0.0001).

***Hypothermia following eOGD-R preserved ATP downregulation more efficiently in neurons than astrocytes.*** Decades of mechanistic research in therapeutic hypothermia suggests that multiple complex targets are involved in hypothermia’s mechanism(s) of action. Nonetheless, in neurons and astrocytes, the effects of hypothermia on ATP production after eOGD-R remain elusive. The hypothermia protocol (31.5 °C), which started along with reperfusion for 20 h, increased ATP production in both cells; however, the gap in ATP production between normothermia and hypothermia was more accentuated in neurons than in astrocytes (Figure 2A,B, *p*-value ≤ 0.0001). In neurons, hypothermia buffered ATP downregulation by a 1.21-fold decrease, while normothermia was associated with a 1.56-fold decrease (Figure 2A, *p*-value ≤ 0.0001), In astrocytes, hypothermia prevented ATP output drop by a 1.10-fold decrease, while normothermia caused a fold decrease by 1.21 (Figure 2B, *p*-value ≤ 0.0001). In comparison, hypothermia prevented ATP deficit more efficiently in neurons than astrocytes (Figure 2A,B, *p*-value ≤ 0.0001). 

**Figure 3:**
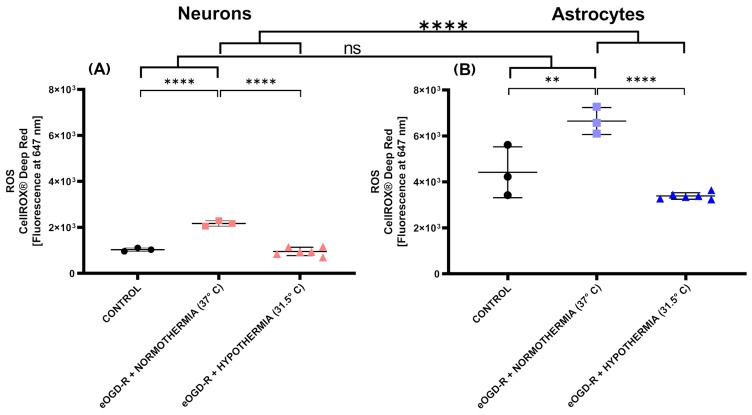
(**A**) In neurons, normothermic 6 h ischemia and 20 h reperfusion protocol in vitro (eOGD-R) caused significant reactive oxygen species (ROS) production versus the control (*p*-value ≤ 0.0001). Hypothermia treatment (31.5 °C) for 20 h remarkably prevented ROS burst against eOGD-R insult, as shown by ROS downregulation compared with the normothermia group (*p*-value ≤ 0.0001). (**B**) In astrocytes, eOGD-R caused significant ROS production versus the control (*p*-value ≤ 0.01). Hypothermia treatment (31.5 °C) remarkably prevented ROS burst against eOGD-R insult, as shown by ROS downregulation compared with the normothermia group (*p*-value ≤ 0.0001). Compared with astrocytes, neurons had a non-significant positive trend on ROS levels after normothermia eOGD-R ((**A**,**B**) *p*-value = 0.17), while hypothermia in neurons after eOGD-R resulted in drastic ROS downregulation. In astrocytes, hypothermia after eOGD-R caused, on average, lower ROS production than its control, but was not statistically significant (*p*-value = 0.09). Hypothermia had a more evident effect downregulating ROS generation in neurons compared to astrocytes (*p* ≤ 0.0001).

***Compared with astrocytes, oxidative stress generation in neurons had a non-significant positive trend after eOGD-R.*** A large body of data shows that the generation of free radicals is a crucial aspect of IRI pathophysiology. However, the characterization of reactive oxidative species (ROS) in neurons and astrocytes following eOGD-R is not well-known. Employing a fluorescent, global ROS probe, we studied general oxidative burst in neurons and astrocytes after eOGD-R. While eOGD-R significantly increased ROS output in both neurons and astrocytes, ROS upregulation in neurons had a non-significant positive trend versus astrocytes, with a fold increase of 2.11 and 1.50, respectively (Figure 3A,B, *p*-value = 0.17). Of note, the power analysis indicated that the positive trend would reach statistical significance with a higher *n*, assuming relative preservation of standard deviations.

After eOGD-R, hypothermia exerted a potent effect in preventing ROS production, with a more pronounced impact observed in neurons compared to astrocytes. A body of preclinical studies demonstrated that hypothermia has a robust effect preventing free radical generation after IRI. However, little is known about hypothermia’s differential actions on neurons and astrocytes following eOGD-R. Our fluorescent probe measuring global ROS revealed that in neurons and astrocytes, hypothermia downregulated free radical production after eOGD-R. In fact, in both cell lines, the average ROS value was low enough to present non-statistical significance between the hypothermia and control groups (Figure 3A,B). In contrast with the normothermia groups, hypothermia in neurons decreased ROS output by 2.27-fold, while in astrocytes, such a fold decrease was by 1.96 (Figure 3A,B, *p*-value < 0.0001).

**Figure 4:**
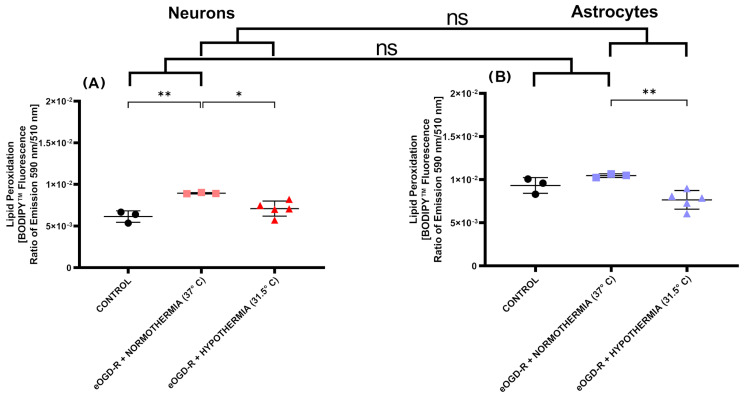
(**A**) In neurons, normothermic 6 h ischemia and 20 h reperfusion protocol in vitro (eOGD-R) caused significant lipid peroxidation (LPO) output versus the control (*p*-value ≤ 0.01). Hypothermia treatment (31.5 °C) for 20 h downregulated LPO generation compared to the normothermia group following eOGD-R insult (*p*-value ≤ 0.05). (**B**) In astrocytes, 6 h + 20 h eOGD-R did not cause a statistically significant increase in LPO production (*p*-value = 0.31). Hypothermia treatment (31.5 °C) for 20 h significantly downregulated LPO after eOGD-R (*p*-value ≤ 0.01). Of note, hypothermia in astrocytes after eOGD-R decreased LPO at average values below the control, but was not statistically significant (*p*-value = 0.0790). In conclusion, LPO was significantly upregulated after normothermic eOGD-R in neurons but not in astrocytes. In both cells, hypothermia after eOGD-R significantly downregulated LPO compared with the normothermia eOGD-R groups. Hypothermic eOGD-R downregulated LPO in astrocytes, causing on average a non-significant trend below its control. No statistically significant differential effect was observed on how hypothermia after eOGD-R affected LPO output in neurons and astrocytes (*p*-value = 0.27).

***Normothermic eOGD-R significantly increased lipid peroxidation in neurons, and not in astrocytes***. ROS produced during IRI react with polyunsaturated fatty acids of cell membranes, inducing lipid peroxidation (LPO). Usually, the LPO measurement is used as a surrogate marker of ROS; however, there is a gap in the literature on how eOGD-R affects LPO output in cell cultures of neurons and astrocytes. Our fluorescent probe specific for LPO revealed that following eOGD-R, LPO significantly increased in neurons after eOGD-R (Figure 4A, 1.45-fold increase, *p*-value ≤ 0.01). In astrocytes, the 1.12-fold increase was not statistically significant (Figure 4B, *p*-value = 0.31). 

***Hypothermia after eOGD-R downregulated lipid peroxidation in both neurons and astrocytes; however, no differential effect was observed between both cell types, and a non-statistically significant “trend” of excessive downregulation was present only in astrocytes.*** Therapies capable of downregulating LPO after eOGD-R are deemed as beneficial; however, since ROS are involved in cell signaling, free radical scavenger production, metabolic modulation, and other complex cellular processes, an excessive detriment in their production can result in unintended effects. Hypothermia significantly decreased LPO generation versus normothermic eOGD-R control by 1.36-fold (Figure 4B, *p*-value ≤ 0.01), while the non-statistically significant decreases versus the astrocyte controls were equal to 1.21-fold (Figure 4B, *p*-value = 0.0790). No statistically significant discrepancy was found in how hypothermia following eOGD-R influenced LPO output between the neurons and astrocytes (*p*-value = 0.27). 

**Figure 5:**
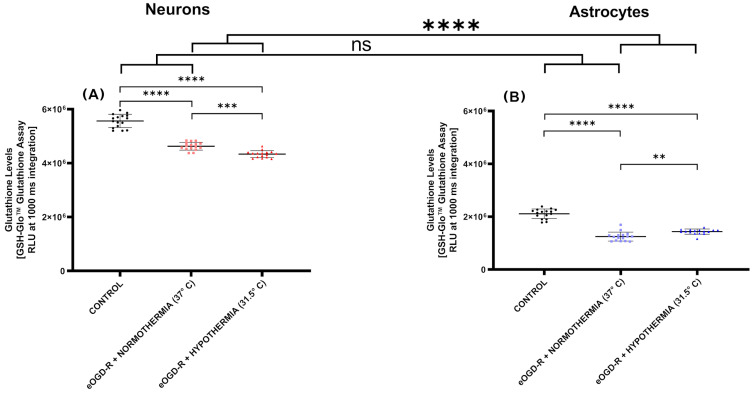
(**A**) In neurons, normothermic 6 h ischemia and 20 h reperfusion protocol in vitro (eOGD-R) caused significant depletion of glutathione (GSH) output versus the control (*p*-value ≤ 0.0001). Hypothermia treatment (31.5 °C) for 20 h further decreased GSH production versus the controls and normothermia eOGD-R (*p*-value ≤ 0.0001 and ≤ 0.001, respectively). Units expressed as relative light units (RLUs). (**B**) In astrocytes, 6 h + 20 h normothermia eOGD-R significantly downregulated the GSH levels (*p*-value ≤ 0.0001). Therapeutic hypothermia (31.5 °C) for 20 h significantly upregulated GSH versus normothermia eOGD-R (*p*-value ≤ 0.01). Although no differential effect was observed between neurons and astrocytes following eOGD-R, hypothermia treatment induced a significant and contrasting effect on GSH production (*p*-value ≤ 0.0001). Units expressed as RLU.

***eOGD-R downregulated glutathione output in neurons and astrocytes; however, no significant differential effect was observed between cell types.*** Glutathione (GSH) is considered one of the most powerful free radical scavengers, and thus has high relevance in the setting of IRI. Although GSH was significantly downregulated in both neurons and astrocytes after eOGD-R, no statistically significant differential effect was observed between both cell types compared with their respective controls (Figure 5A,B, *p*-value = 0.44). Following eOGD-R, GSH was decreased by 1.69-fold in astrocytes, while in neurons, it was decreased by 1.20-fold (Figure 5A,B, *p*-value ≤ 0.0001).

***Hypothermia after eOGD-R further decreased glutathione in neurons, while in astrocytes, hypothermia upregulated the glutathione levels.*** Animal models of cardiac arrest demonstrated that hypothermia increases GSH levels in the blood and cerebrospinal fluid; however, little is known about hypothermia’s specific effect on neurons and astrocytes after eOGD-R. Compared with normothermia eOGD-R, our eOGD-R + hypothermia protocol revealed a significant decrease in GSH levels in neurons by 1.06-fold (Figure 5A, *p*-value ≤ 0.0001), while in astrocytes, the GSH levels were significantly increased by 1.15-fold (Figure 5B, *p*-value ≤ 0.01). This broad and opposite differential effect of eOGD-R + hypothermia on neurons and astrocytes was found to be statistically significant (Figure 5A,B; *p*-value ≤ 0.0001) 

## 4. Discussion

Hypothermia as a medical treatment was documented in the *Edwin Smith Papyrus*, which is the oldest medical literature known to date [17]. Since the first guidelines endorsing hypothermia’s application as a neuroprotective agent after cardiac arrest, many experimental studies have tested different hypotheses exploring its mechanisms [18]. Among these, mitochondria functioning preservation, modulation of oxidative stress, and cytoprotection are some of the most prominent targets [19]. However, there is a gap in the knowledge of how ischemia-reperfusion injury and hypothermia specifically affect certain key cells respective to brain functioning such as neurons and astrocytes. In light of recent discoveries on mitochondria transference between neurons and astrocytes, understanding these putative specificities represents a foundational stone for the development of novel therapies with enhanced cytoprecision [14,20,21].

In our current study, eOGD-R caused a higher leakage of LDH in neurons than in astrocyte cultures; however, cytoprotection from hypothermia, as per the LDH levels, occurred only in neurons (Figure 1). Our findings on this differential neuronal-astrocytic capacity to tolerate IRI insults have been described by previous studies [22,23]. Although astrocytes are generally considered more resistant than neurons to damage by insults like eOGD-R, astrocytes exhibit greater sensitivity than neurons to high concentrations of LDH and acidosis [24,25]. Consequently, astrocytes are equipped with specific isoforms of the monocarboxylic acid transporter (MCT), which are optimized to facilitate lactate flux. In contrast, MCTs expressed in neurons serve as a barrier to increased transport when lactate levels increase above 1 mM. Furthermore, it has been documented that astrocytes can use lactate as an energetic substrate for oxidation after ischemia. However, there is no evidence of similar capabilities by neurons, which may explain why after the same eOGD-R insult, neurons generate higher lactate levels [22]. To our knowledge, no studies have measured the LDH levels in astrocytes after eOGD-R (6 + 20 h) and hypothermia (31.5 °C ~20 h). However, in contrast to the neutral effect of hypothermia on astrocytes that we found, a study with a hypoxia model showed reduced LDH levels in rat primary astrocytes after a 24 h hypothermia protocol at 32 and 34 °C [26]. These unequal experimental results can be caused by a multitude of different variables in the two different models including the hypoxia-only insult versus our full eOGD-R protocol.

In our experiments, eOGD-R caused stronger ATP depletion in neurons compared with astrocytes, nonetheless, hypothermia buffered ATP deficit more effectively in neurons (Figure 2). ATP measurement has been validated as a marker of cell viability by numerous studies, and ATP depletion is unanimously deemed as a key aspect of ischemic injury, given the subsequent influx of calcium and consequent ROS generation by mitochondrial depolarization [27,28,29,30,31]. The higher resilience to ischemia-reperfusion injury shown by the astrocytes in comparison to neurons has been experimentally proven; in 1993, Goldberg and Choi showed that while neurons were irreversibly damaged after 60–70 min of OGD, astrocytes were comparably injured only after past 4 h of sustained OGD [32,33]. Accumulating evidence further reveals that the remarkable survival of astrocytes is related with specific phenotypic characteristics; astrocytes are not only capable of anaerobic glycolysis upregulation during anoxia, but they can efficiently oxidize different energetic substrates, which are characteristics not displayed by neurons [34,35]. The mild but intriguing difference found in ATP levels after eOGD-R and hypothermia has not been properly studied in comparative studies between neurons and astrocytes. However, it is known that hypothermia after global brain ischemia enhances glutamate uptake, and it has been suggested that this occurs due to hypothermia’s effect on astrocytes, which are the most important brain cells involved in extracellular glutamate clearance [36,37,38]. Additionally, heat shock proteins (HSPs) have been known to be involved in compensatory ATP synthesis pathways during ischemia-reperfusion injury and different studies have documented differential neuronal-astrocytic expression of HSP markers after this insult [39,40]. However, to date, no studies have compared hypothermia’s effect on HSP expression in neurons and astrocytes following eOGD-R. 

In this research initiative, ROS generation displayed a non-statistically significant trend, with neurons exhibiting higher levels compared to astrocytes following normothermic eOGD-R. In neurons, hypothermia caused the most physiological ROS output after eOGD-R insult (Figure 3). The excessive production of ROS is, by general consensus, considered a critical factor in the genesis of ischemia-reperfusion injury [29]. Since the initial description of reoxygenation as a fundamental aspect of reperfusion injury by Hearse et al. in 1973, a body of extensive research has provided novel insights into the enzymatic and cellular origins of ROS after ischemia-reperfusion [41]. However, the discrepancies (and their mechanisms) on ROS generation after ischemia-reperfusion in neurons and astrocytes have not been well-studied. Astrocytes putatively buffer free radical damage better than neurons as they are less dependent on oxidative phosphorylation and have substantially higher levels of glutathione, which plays a pivotal role in ROS scavenging systems [42]. Although ROS output downregulation after hypothermia has been studied in neurons, the effect on astrocytes remains poorly characterized [43]. In our experiments, we observed that in astrocytes, hypothermia caused a trend of ROS generation at lower levels (on average) than the controls. Considering that large clinical trials have shown mixed results after hypothermia treatment, different groups of investigators have suggested that hypothermia can result in protective or toxic effects depending on different factors such as temperature goal and duration, cooling method, reperfusion status, and type of neurovascular unit [44,45]. In fact, recent data have shown that astrocytes can express protective or toxic phenotypes when they become reactive [46,47]. Further research will be required to determine whether ROS downregulation below the controls is associated with unfavorable or toxic astrocytic phenotypes.

Additionally, we found that normothermic eOGD-R significantly increased lipid peroxidation in neurons, but not in astrocytes. While HPT after eOGD-R downregulated lipid peroxidation in both neurons and astrocytes, a non-statistically significant trend of “excessive downregulation” was present only in astrocytes (Figure 4). Cell membranes are constituted by different lipids including polyunsaturated fatty acids. Free radicals can react and oxidize these fatty acids, forming lipid hydroperoxides and an alkyl radical [48]. The phenomenon of lipid peroxidation impairs normal cell membrane configuration, compromising its fluidity and integrity [49]. Therefore, lipid peroxidation is usually quantified as an indirect, surrogate marker of ROS effects on cellular targets. Although increased lipid peroxidation in neurons has been described after OGD-R by other groups, to our knowledge, no comprehensive study has described lipid peroxidation changes in astrocytes after eOGD-R and hypothermia [50]. Very interestingly, the excessive downregulation in lipid peroxidation observed only in astrocytes exhibited the same pattern as in the general ROS measurement (Figure 3). Since ROS constitute a complex, dynamic, and fluid system involved in bioenergetics, cell death, and signaling, downregulation beyond the controls after eOGD-R and hypothermia treatment should not be deemed as a desirable effect per se.

Noteworthy, in our study, eOGD-R downregulated glutathione output in both cells; however, no differential effect was observed between neurons and astrocytes after the insult. Hypothermia after eOGD-R further decreased glutathione in neurons, while in astrocytes, hypothermia upregulated the glutathione levels, revealing a significant and contrasting differential response from this treatment (Figure 5). Glutathione’s importance has to be highlighted in the context of ischemia-reperfusion injury and free radical generation, considering that it is the most important antioxidant synthesized by cells [51]. Almeida et al. published a similar study on primary cells, applying a 1 h OGD only protocol (no reperfusion) and found that glutathione suffered oxidation in neurons but not in astrocytes, further supporting our previous statements on the increased neuronal susceptibility to ischemic damage [52]. The lack of reperfusion (OGD vs. eOGD-R), short OGD duration (1 h vs. 6 h), incomplete nutritional depletion (OGD vs. eOGD-R), and cell culture type (primary cells vs. cell line) may account for the observed differences in our experiments. Furthermore, the study by Almeida et al. found a clear correlation between electron transport chain impairment and mitochondrial generalized dysfunction with the damage caused in neurons after OGD, which was not observed in astrocytes [52]. These findings further support the importance of mitochondria function and dysfunction when assessing the differential susceptibilities between neurons and astrocytes after in vitro ischemia-reperfusion. The current knowledge on glutathione changes after eOGD-R and hypothermia is scarce. A study on cardiac arrest in rabbits published by Zhao and Chen showed that 24 h hypothermia consistently increased glutathione in the blood and cerebrospinal fluid [13]. However, at the time of writing, no studies have comprehensively characterized glutathione modifications in neurons and astrocytes after eOGD-R and hypothermia. The fact that hypothermia causes opposite changes in glutathione levels after eOGD-R suggests, once again, that different brain cells and neurovascular units may be benefited or damaged from the same treatment intervention. Taken together, the available body of scientific evidence suggests that the differential neuronal-astrocytic response following ischemia-reperfusion and hypothermia obeys a significant number of interrelated biological and phenotypic idiosyncrasies including differences in energy expenditure, glutamate excitotoxicity susceptibility, inflammatory response involvement, ROS buffering capabilities, calcium excitotoxicity, programmed cell death thresholds, and mitochondrial transference/exchange, among others [14,53,54,55,56,57,58].

## 5. Conclusions

To the best of our knowledge, this is the first experimental study where ATP, LDH, ROS, lipid peroxidation, and glutathione were concomitantly measured in neurons and astrocytes after eOGD-R and hypothermia. An additional and novel aspect of this study includes the fact that in vitro ischemia-reperfusion was conducted through an enhanced protocol of macronutrient depletion compared with most of the published studies where glucose deprivation was the only nutritional deprivation, which in the authors’ opinion, can misrepresent the biological phenomena observed in global ischemia after cardiac arrest [15,59]. Further research will be required to determine whether a reconciliation (if exists) in the observed differential neuronal and astrocytic effects after eOGD-R and hypothermia is dose, time, and modality dependent [60]. ***“All substances are poisons; there is none which is not; the dose differentiates a poison from a remedy” Paracelsus***.

## 6. Limitations

Although hypoxia and nutritional deprivation are well-established models of in vitro ischemia-reperfusion injury, it must be stated that isolated cell lines in a Petri dish are not necessarily equal to primary cells or in vivo biological conditions [15,61,62]. In fact, it has been documented that culturing conditions may affect gene and receptor expression, which restricts the extrapolation to in vivo conditions [63,64]. Furthermore, it should be acknowledged that our findings do not necessarily apply to all neurons and astrocytes, since in normal biology, cells exhibit substantial heterogeneity, presenting different phenotypes and therefore potentially unequal responses to insults and treatments [33]. However, cell cultures offer a number of remarkable advantages when studying ischemia-reperfusion injury including the capacity to understand metabolic phenomena in a specific cell type due to cell population homogeneity, the possibility of exploring the specific impact of substrate versus oxygen versus combined substrate–oxygen deprivation as well as the opportunity to easily modify variables (pH, ion, and media composition), among others [22]. Finally, the preliminary studies in this publication do not describe the precise mechanisms on the observed differential susceptibility between neurons and astrocytes after eOGD-R and hypothermia. 

## Data Availability

The datasets generated in the current study are not publicly available due to pending grant applications. However, they are available from the corresponding author on reasonable request.
